# Hikikomori as a possible clinical term in psychiatry: a questionnaire survey

**DOI:** 10.1186/1471-244X-12-169

**Published:** 2012-10-15

**Authors:** Masaru Tateno, Tae Woo Park, Takahiro A Kato, Wakako Umene-Nakano, Toshikazu Saito

**Affiliations:** 1 Department of Neuropsychiatry, Sapporo Medical University, School of Medicine, South-1, West-16, Chuo-ku, Sapporo 0608543, Japan; 2 Department of Psychiatry, Boston University School of Medicine and VA Boston Healthcare System, 251 Causeway Street, Boston, MA, 02114, USA; 3 Department of Neuropsychiatry, Graduate school of Medical Sciences, Kyushu University, 3-1-1 Maidashi Higashi-ku, Fukuoka, 8128582, Japan; 4 Department of Psychiatry, School of Medicine, University of Occupational and Environmental Health, Iseigaoka, Yahatanishi-ku, Kitakyushu, Fukuoka, 8078555, Japan

**Keywords:** Hikikomori, Social withdrawal, School refusal, Psychiatric diagnosis, Developmental disorders

## Abstract

**Background:**

The word hikikomori, the abnormal avoidance of social contact, has become increasingly well-known. However, a definition of this phenomenon has not been discussed thoroughly. The aim of this study is to gain a better understanding of the perception of hikikomori amongst health-related students and professionals and to explore possible psychiatric conditions underlying hikikomori.

**Methods:**

A total of 1,038 subjects were requested to complete a questionnaire regarding hikikomori phenomenon.

**Results:**

While some differences in the perception of hikikomori do exist, all subjects tended to disagree with the statement, “hikikomori is NOT a disorder”. Regarding the underlying psychiatric disorders of hikikomori, approximately 30% of psychiatrists chose schizophrenia as the most applicable ICD-10 diagnosis for hikikomori, whereas 50% of pediatricians chose neurotic or stress-related disorders.

**Conclusions:**

An argument still exists regarding the relationship between hikikomori and psychiatric disorders. We propose that the term hikikomori could be used to describe severe social withdrawal in the setting of a number of psychiatric disorders.

## Background

The word hikikomori was recently added to the Oxford Dictionary of English [1] where it joins other words of Japanese origin such as otaku (person with obsessive interests) and karoshi (death from overwork). It is defined as ‘the abnormal avoidance of social contact, typically by adolescent males’. Hikikomori was first introduced to the public when a Japanese psychiatrist, Tamaki Saito, published a book with this word in its title in 1998 [2]. In his book “Social Withdrawal (*shakaiteki hikikomori*): A Neverending Adolescence”, Saito defined hikikomori provisionally as ‘those who withdraw entirely from society and stay in their own homes for more than six months, with onset by the latter half of their twenties, and for whom other psychiatric disorders do not better explain the primary causes of this condition’. Since then, the word hikikomori has been used widely in Japan and has more recently been reported in the foreign media and discussed in medical journals by psychiatrists from other countries [3-13]. Much of this attention occurred without a thorough discussion of its precise definition [14-17].

In May 2010, a research group supported by the Japanese government published guidelines for the assessment and treatment of hikikomori [18]. The guidelines defined hikikomori as the following: ‘A phenomenon in which persons become recluses in their own homes, avoiding various social situations (e.g. attending school, working, having social interactions outside of the home etc.) for at least six months. They may go out without any social contact with others. In principle, hikikomori is considered a non-psychotic condition distinguished from social withdrawal due to positive or negative symptoms of schizophrenia. However, there is a possibility of underlying prodromal schizophrenia’.

These guidelines were based on a number of studies including the analysis of 184 consecutive hikikomori cases at five different mental health centers (Kondo’s survey cited in the guidelines [18]; unpublished), the investigation of hikikomori youths referred to psychiatric emergency services (Nakashima’s survey cited in the guidelines [18]; unpublished), and a nationwide survey conducted as a part of the WHO World Mental Health Initiatives (WMH-J) [19]. The WMH-J survey selected subjects from voter registration lists in various parts of Japan, interviewed a total of 4,134 respondents aged 20–49 (response rate: 55.1%) and demonstrated that a total of 1.2% had experienced hikikomori. They estimated that there are 232,000 ongoing hikikomori cases in Japan. Furthermore, in September 2010, the Cabinet Office of the Japanese government published results of their study on hikikomori and reported that the number of hikikomori was estimated to be 236,000 [20]. These results demonstrate that hikikomori is a common problem in Japan.

In this study, we conducted a questionnaire survey of hikikomori with 1,038 subjects. Because psychiatrists and pediatricians tend to have the most clinical contact with hikikomori individuals, we included additional questions regarding potential underlying psychiatric conditions of hikikomori for the psychiatrists and pediatricians participating in the study. The overall aim of this study is to gain a better understanding of the perception of hikikomori amongst health-related students and professionals and to explore possible psychiatric conditions underlying hikikomori.

## Methods

### Subjects

The subjects of this study were psychiatrists who work at one of three University hospitals (Sapporo Medical University, Kyushu University, and the University of Occupational and Environmental Health) or their affiliating hospitals, pediatricians who are on the electronic mailing list of the Japanese Society of Psychosomatic Pediatrics, clinical psychologists who are included in the electronic mailing list of the Society of Certified Clinical Psychologists in the Hokkaido region of Japan, nurses of all 25 different clinical services at Sapporo Medical University Hospital, students at Sapporo Medical University including the School of Medicine and the School of Health Sciences, and others who received invitations to this study through the above-mentioned subjects. The three universities were selected as a study site because three of the authors (MT, TAK and WUN) worked together for previous studies [21,22]. These study subjects were chosen primarily to increase the response rate with the belief that health-related students and professionals would have greater interest in this area of study and that these subjects would be more responsive to the survey request. Demographics of study subjects other than profession were not collected.

The total number of the respondents was 1,038 and a description of the respective groups is shown in Table 1. Respondents were divided into five different groups: medical doctors (psychiatrists and pediatricians), nurses, psychologists, students and others. The response rate of each group was as follows: psychiatrists 85.1%, nurses 89.7% and students 79.5%. Because two mailing lists used in this study contained a number of invalid addresses, it was difficult to verify response rates of pediatricians and clinical psychologists. However, based on the report by each mailing list manager, the response rate was estimated to be 60 percent for both groups.

**Table 1:** The summary of the results

	**Medical doctor (n=106)**	**Nurse (n=595)**	**Psychologist (n=46)**	**Student (n=229)**	**Others (n=62)**
	**(psychiatrist / pediatrician) (n=80 / n=26)**				
To be diagnosed as hikikomori, how long do they withdraw from the society and keep staying in their home?	6.32±3.7 (6.85±3.9 / 4.69±2.3)	3.83±3.8	5.67±4.1	2.95±3.4	3.82±2.5
Hikikomori is NOT a disorder	2.70±1.1 (2.74±1.0 / 2.58±1.3)	2.61±1.1	2.80±1.0	2.86±1.1	2.81±1.2
Hikikomori persons shut themselves in their room the while day and rarely see their family	3.22±1.2 (3.20±1.2 / 3.27±1.2)	3.73±1.2	3.11±1.2	3.74±1.2	3.35±1.2
Hikikomori individuals sometimes go out for grocery stores and bookstores	3.52±1.1 (3.57±1.1 / 3.42±1.1)	2.91±1.3	3.61±1.1	2.86±1.2	3.33±1.2
School refusal closely relates to hikikomori	4.12±0.9 (4.11±0.9 / 4.15±1.0)	3.99±1.0	3.89±0.9	3.90±1.1	3.95±1.0
Recovery is possible in individuals with hikikomori	3.78±0.8 (3.67±0.8 / 4.04±0.7)	4.00±0.9	4.13±0.8	4.26±0.8	4.08±0.9
Hikikomori is related to biological factors such as psychiatric disorders or developmental disorders	3.87±0.9 (3.86±0.8 / 3.85±1.1)	3.30±1.0	3.70±0.8	3.17±1.0	3.48±0.9
Hikikomori is related to psychological factors such as bullying at school or frustration at workplace	3.99±0.8 (3.91±0.7 / 4.23±0.9)	4.24±0.7	4.02±0.6	4.39±0.7	4.16±0.6
Hikikomori is related to social factors such as family environment which accepts the situation	4.02±0.7 (3.96±0.7 / 4.19±0.7)	4.03±0.8	3.85±0.7	4.16±0.8	3.87±0.7
Hikikomori is related to Japanese society, culture or national characteristics	3.61±1.0 (3.65±0.9 / 3.50±1.2)	3.16±1.1	3.30±0.9	3.40±1.0	3.18±1.0

The results are expressed as the mean ± standard deviation (SD). On the five-point Likert scale, score 5 means the strong agreement.

### Methods of data collection

The study authors created an online questionnaire and e-mailed the web link and login password for the questionnaire page to collaborators. The site collaborators then distributed the invitation e-mail to their colleagues. For nurses and students, a printed questionnaire was distributed. Both the online and printed questionnaires took approximately 10 minutes to complete.

All subjects were requested to complete the questionnaire within the survey period between May 1 and July 31, 2010.

### Questionnaire

The questionnaire contained three types of responses: open responses (the length of social withdrawal), responses on a five-point Likert scale and single choice responses (psychiatric diagnosis) (Table 1). A Likert scale is a commonly used method for the measurement of attitudes in survey studies [23,24]. The subjects were asked to rate their answers to a statement using a five-point scale ranging from one, indicating strongly disagree, to five, indicating strongly agree, with three representing a neutral response that neither agreed nor disagreed with the statement. Additionally, psychiatrists and pediatricians were asked what they felt would be the most applicable psychiatric diagnosis of hikikomori from the F codes (blocks) in Chapter V of the ICD-10 [25] (Table 2).

**Table 2:** Psychiatric background of hikikomori

**ICD-10 Chapter V**	**Psychiatrists**	**Pediatricians**
	**(n=80)**	**(n=26)**
F2: Schizophrenia, schizotypal and delusional disorders	30.0%	3.8%
F3: Mood (affective) disorders	2.5%	11.5%
F4: Neurotic, stress-related and somatoform disorders	16.3%	50.0%
F6: Disorders of adult personality and behavior	21.3%	3.8%
F7: Mental retardation	2.5%	0%
F8: Disorders of psychological development	17.5%	23.1%
Hikikomori is not a disorder	1.3%	0%
No idea	8.8%	7.7%

Psychiatrists and pediatricians were requested to select the most applicable psychiatric diagnosis of their experienced hikikomori cases according to the ICD-10.

### Ethical matters

The aim of this study was clearly stated on the online survey system’s main web page and the cover sheet of the printed version. Completing the questionnaire was deemed to constitute consent. All respondents participated in this study without any incentive provided by the study investigators. Similarly, all authors and subjects involved in this study declared themselves free of any conflict of interest relating to the present study. The study protocol (Shakaiteki Hikikomori Ni Kansuru Kenkyu) was approved by the ethics committee of Sapporo Medical University, and this study was conducted in compliance with the Helsinki Declaration.

## Results

The results of the study are summarized in Table 1. Responses to the question regarding the length of social withdrawal necessary for diagnosis of hikikomori showed a great diversity, ranging from the longest of 6.85±3.9 months by psychiatrists to the shortest of 2.95±3.4 months by students. In response to many statements, answers revealed a certain amount of consistency. When asked to respond to the statement, “hikikomori is NOT a disorder”, all five groups scored less than three, suggesting that all five groups tended to disagree with the statement. The lowest score was made by nurses (2.61±1.1) and the highest by students (2.86±1.1). The typical image of hikikomori involving someone who shuts themself in their room and rarely looks at other family members in their homes tended to be agreed with (all five groups answered higher than three). The statement ‘hikikomori individuals sometimes go out for grocery stores and bookstores’ was agreed to on average by psychiatrists, clinical psychologists and the “others” group but nurses and students on average disagreed. The responses to the statement that hikikomori is closely related to school refusal showed strong agreement amongst all five groups; from 3.89±0.9 by clinical psychologists to 4.12±0.9 by medical doctors. The idea that recovery from hikikomori is possible also tended to be agreed with though interestingly, the lowest score was observed among psychiatrists (3.67±0.8) while the rest of the groups scored higher than four.

We asked psychiatrists and pediatricians what they felt would be the most applicable psychiatric diagnosis of hikikomori according to the ICD-10 [25], with the option of choosing ‘it is not a psychiatric disorder’ and ‘unsure’. The results are shown in Table 2. 30% of psychiatrists answered schizophrenia would be the most applicable psychiatric diagnosis for hikikomori cases whereas half of the pediatricians chose neurotic, stress-related and somatoform disorders which includes anxiety disorders such as social anxiety disorder or adjustment disorder. It should also be noted that both psychiatrists and pediatricians answered that almost one fifth of hikikomori individuals could be diagnosed as having developmental disorders.

## Discussion

The goals of this study were two-fold: to gain a better understanding of the public’s perception of hikikomori, particularly in health care professionals, and to investigate the difference in the possible underlying psychiatric conditions of hikikomori individuals.

### The perception of hikikomori amongst study participants

Overall, the study participants tended to agree on many responses. This includes opinions on hikikomori regarding school refusal, psychological factors, family environment, and whether or not hikikomori is a disorder or is related to Japanese society. Prior to the Japanese government’s publication of the new hikikomori guidelines in 2010, there was no official definition of hikikomori. The hikikomori phenomenon before that time was described initially by Saito’s book and was often cited as if it described formal diagnostic criteria. Additionally, the idea of hikikomori was spread in popular media. Thus it is not surprising that many respondents had similar opinions about hikikomori.

Some differences in opinion did exist in the results of the questionnaire. Psychiatrists and psychologists tended to group together in their differences in opinion. They generally felt that the length of social withdrawal felt to be sufficient for a hikikomori diagnosis was longer, 6.85±3.9 months in psychiatrists and 5.67±4.1 in psychologists. In contrast to this result, students on average felt that much less time was needed for a diagnosis of hikikomori (2.95±3.4 months), the most popular answer being one month (n=96, 41.9%). These results indicate that other respondents had a lower threshold for what is considered an abnormal amount of social withdrawal. The opinion that ‘hikikomori individuals sometimes go out to grocery stores and bookstores’ was viewed differently by the respective groups. Psychiatrists (3.52±1.1) and psychologists (3.61±1.1) tended to agree with this view. Nurses (2.91±1.3) and students (2.86±1.2) tended to disagree, suggesting that their image of an individual with hikikomori as someone who spends most of their time confined to his/her own room. Psychiatrist and psychologists, as well as pediatricians, also tended to feel more strongly that hikikomori was related to biological factors such as underlying psychiatric or developmental disorders than the other respondents. Of note, both questions regarding occasional leaving of one’s room and underlying psychiatric diagnoses represent two significant differences between the definition in the new guidelines and that of Saito’s book in 1998. The definition in the new guidelines allows for leaving one’s home as long as it is without social interaction and refers to the difficulties of excluding prodromal schizophrenia. The changes in the guidelines might account for the differences seen in the respondents on these two issues.

The final notable difference in opinions was observed in responses to the statement ‘recovery is possible in individuals with hikikomori’. Though the average response of most groups was four or five, psychiatrists on average scored lower than four (3.67±0.8). It is possible that some individuals examined in psychiatric clinics were diagnosed with schizophrenia by psychiatrists. Such cases might be perceived as having a greater difficulty in overcoming their clinical symptoms.

Regarding the issue of school refusal, this criterion is well known to be closely related to hikikomori [2]. Defined by the Ministry of Education, Culture, Sports, Science & Technology in Japan, school refusal is a protracted absence from school, typically more than 30 days per year, caused by psychological factors such as fear, anxiety, anger, and sense of refusal [26]. These feelings can cause emotional conflict and guilt about staying at home. This conflict commonly presents with somatic complaints. In the present study, all five groups agreed with the statement that ‘school refusal closely relates to hikikomori’, suggesting the importance of school refusal in the concept of hikikomori. A follow-up study of students who refuse to go to school by Saito et al. revealed that about ten percent of them evolve into hikikomori during their youth [18]. Based on this data, the new hikikomori guidelines recommend early intervention in school refusal in order to prevent development of hikikomori.

The biopsychosocial model is a well-known model which describes that biological, psychological, and social factors, all play an important role in psychiatric conditions [27]. In this study, biological factors include psychiatric disorders and developmental disorders, psychological factors include difficulty in school such as being bullied, taunted, and refused by school peers, or a sense of failure at their workplace, and social factors contain environmental factors such as excessively protective parenting style and dysfunctional family dynamics. The results in this study suggest that medical doctors regarded biological factors as more important compared to other groups, while all groups thought that both psychological and social factors were related to hikikomori.

In the final question of the survey, respondents tended to believe that hikikomori is related to Japanese culture and society. An international questionnaire survey by Kato et al. found that Japanese psychiatrists tended to believe social and cultural factors played a larger role in hikikomori than psychiatrists from other countries, and tended to be more passive in providing medical intervention in hikikomori cases [15]. This passivity may be related to *amae*, a Japanese word that describes dependent behaviors typically observed between parents and their children, one of the factors believed to contribute to the Japanese cultural acceptance of this phenomenon [28]. Individuals with a sense of *amae* may beg or plead, behave selfishly and indulgently, while secure in the knowledge that the caregiver will forgive their acts. This concept of *amae* might cause delay in help-seeking behavior of family members of hikikomori subjects.

### Underlying psychiatric disorders of hikikomori

Regarding the question of underlying psychiatric disorders in hikikomori, there is an ongoing debate amongst parties consisting of three different views; 1) most hikikomori cases can be diagnosed using existing ICD [25] or DSM [29] diagnostic criteria, 2) hikikomori is not a psychiatric disorder and 3) hikikomori represents a new diagnostic category [30]. Kondo and his group reported that most hikikomori cases can be diagnosed using current diagnostic criteria [31]. One interpretation of the second view is that hikikomori is a culture-bound syndrome [17]. The above debate raises questions about whether hikikomori is a mental illness by itself. Though a ‘case’ of hikikomori may be identifiable, ‘caseness’ does not necessarily guarantee the presence of mental illness [32] and in the case of hikikomori, may imply the presence of numerous illnesses or none at all.

In this study, respondents tended to believe that hikikomori individuals could be diagnosed with existing psychiatric diagnoses, specifically the F codes in chapter V of the ICD-10 [25]. Approximately 30% of psychiatrists chose F2 (Schizophrenia, schizotypal and delusional disorders), the most commonly chosen diagnosis by psychiatrists. The most commonly chosen diagnosis amongst pediatricians at 50% was F4 (Neurotic, stress-related and somatoform disorders). This difference in opinion might be explained by the difference in age of the patients typically seen at their respective clinics.

It is noteworthy that both psychiatrists and pediatricians answered that almost one fifth of their hikikomori patients could be diagnosed with F8 (Disorders of psychological development) such as pervasive developmental disorders (PDD). Kondo’s survey cited in the new guidelines [18] reports that among hikikomori cases who visited mental health centers in five different prefectures in Japan, about 30% of them were diagnosed as having developmental disorders . Recent epidemiological studies demonstrate that the number of individuals with the diagnosis of PDD is rising [33-35]. The criteria for the diagnosis of PDD such as autism and Asperger's syndrome include: 1) qualitative impairment in social interaction and 2) restricted, repetitive, and stereotyped patterns of behavior, interests, and activities. Most hikikomori cases have difficulties in reciprocal social interaction. Thus, a co-morbid diagnosis of PDD should be considered in individuals diagnosed with hikikomori and a careful developmental history should be taken.

The results of the present study were similar to the results of previous epidemiological studies in regards to the underlying psychiatric disorders of hikikomori [31]. In an investigation of hikikomori youth (under 30 years old) who were referred to psychiatric emergency services, the proportion of the diagnosis of psychiatric disorders coded in F2, F4 and F8 was almost equal (Nakashima’s survey cited in the guidelines [18]; unpublished). In a different study, psychiatric disorders were observed in 125 cases of severe social withdrawal. 27% of these cases were diagnosed with developmental disorders such as PDD [30]. Other common diagnoses included anxiety disorders (22%), personality disorders (18%), mood disorders (14%) and psychosis (8%). In this study, it was concluded that hikikomori cases could be divided into three groups: 1) patients with diagnoses such as schizophrenia, mood disorders, anxiety disorders who might respond well to biological interventions (i.e., psychopharmacotherapy), 2) patients with developmental disorders such as PDD or mental retardation for whom a more psychological approach including social skills training, vocational training and utilization of social resources are needed, and 3) patients with personality disorders, distortion of personality trait or identity problems and who may have comorbid psychiatric disorders e.g. mood disorders or anxiety disorders for whom psychotherapy, social and vocational support should be performed. Regarding subjects categorized in the third group, it was warned that they may not respond well to pharmacotherapy despite a primary diagnosis of mood disorder or anxiety disorder.

### Study limitations

There were several limitations in this study. First, though the total number of subjects in this study was higher than other previous surveys on hikikomori, we only drew from people with health-related professions, smaller numbers of subjects were studied within some professional subcategories, and we sampled within only a few areas of Japan making it difficult to generalize the results. Second, we did not gather subjects’ demographic information such as age and gender which may have helped to make inferences from the data. Additionally we did not gather data on past history of the contact with hikikomori subjects. The clinical and social experiences of the study subjects pertaining to previous contact with hikikomori individuals could potentially have had a large influence on the answers to the questionnaire.

## Conclusion

The new Japanese government guidelines on hikikomori presented a definition of hikikomori and recommended including the essential aspect of school refusal to the definition. This definition describes the phenomenon in which a person becomes a recluse in his/her own home, avoiding all kinds of social situations for at least six months. In children with school refusal, various feelings can be observed that cause emotional conflict, ambivalence and guilt against staying at home without going to school regardless of their superficial problems. It is likely for such children to result in hikikomori. The prevention of hikikomori might be helped with an intervention for school refusal. Support for hikikomori should be multi-dimensional and comprehensive. Underlying psychiatric disorders including developmental disorders and personality disorders should be carefully assessed for and appropriate interventions should be started promptly.

In conclusion, hikikomori describes a phenomenon characterized by severe social avoidance. The findings suggest that cases of hikikomori can potentially be explained by other underlying psychiatric disorders. Thus though a case of hikikomori may not represent a unique mental illness, it potentially could be treated like R codes of the ICD-10 which represent symptoms, signs and abnormal clinical and laboratory findings, not elsewhere classified. More studies are needed to further this discussion, particularly reports from other countries.

## Competing interests

The authors declare that they have no competing interests.

## Authors’ contributions

MT and TWP contributed to the study’s design. MT, TAK and WUN equally contributed to data collection, and had full access to the data. TS supervised this study and manuscript writing. MT drafted the manuscript. TWP assisted with drafting the manuscript. All authors have read and approved this paper.

## Pre-publication history

The pre-publication history for this paper can be accessed here:

http://www.biomedcentral.com/1471-244X/12/169/prepub
